# Effect of Bond-Line Thickness on Fatigue Crack Growth of Structural Acrylic Adhesive Joints

**DOI:** 10.3390/ma14071723

**Published:** 2021-03-31

**Authors:** Yu Sekiguchi, Chiaki Sato

**Affiliations:** Institute of Innovative Research, Tokyo Institute of Technology, 4259 Nagatsuta-cho, Yokohama 226-8503, Kanagawa, Japan; csato@pi.titech.ac.jp

**Keywords:** DCB fatigue tests, adhesive thickness effect, Paris’ law, structural adhesives

## Abstract

With an increasing demand for adhesives, the durability of joints has become highly important. The fatigue resistance of adhesives has been investigated mainly for epoxies, but in recent years many other resins have been adopted for structural adhesives. Therefore, understanding the fatigue characteristics of these resins is also important. In this study, the cyclic fatigue behavior of a two-part acrylic-based adhesive used for structural bonding was investigated using a fracture-mechanics approach. Fatigue tests for mode I loading were conducted under displacement control using double cantilever beam specimens with varying bond-line thicknesses. When the fatigue crack growth rate per cycle, *da*/*dN*, reached 10^−5^ mm/cycle, the fatigue toughness reduced to 1/10 of the critical fracture energy. In addition, significant changes in the characteristics of fatigue crack growth were observed varying the bond-line thickness and loading conditions. However, the predominance of the adhesive thickness on the fatigue crack growth resistance was confirmed regardless of the initial loading conditions. The thicker the adhesive bond line, the greater the fatigue toughness.

## 1. Introduction

Nowadays, adhesives are widely used in many products and have become an indispensable technology owing to several advantages such as design flexibility, bonding dissimilar materials, and a smooth load transfer. As the number of products using adhesive technology increases, improvements in reliability are becoming crucial. Over their long service lives, adhesively bonded joints are exposed to harsh environmental conditions, such as high humidity and temperature, impact loading, vibration, etc.; therefore, durability against these conditions is essential, especially when used in structural parts. For the fatigue testing of adhesive joints, two standards, ASTM D3166 and ISO 9664 [1,2], are available, although these consider shear strength. In terms of fracturing in the joints, crack resistance is also important, and the fracture toughness of the adhesive layer has been investigated experimentally [3,4,5,6,7,8] and numerically [9,10,11,12,13] under static loading conditions. In the case of fatigue crack resistance, there are no standards available. However, fatigue crack growth (FCG) of composites and adhesives is one of the key issues in the aviation industry; therefore, many studies on FCG have been conducted mainly based on empirical correlations in experiments [14,15,16,17,18,19,20,21,22], and some used the Cohesive Zone Model (CZM) for numerical analysis [23,24,25,26]. In the fatigue tests, FCG behavior is examined by relating crack initiation per cycle with a fracture parameter. The relation between the FCG rate and the fracture parameter was first developed for metals as a function of the stress intensity factor K [27], the linear range of which is called the Paris’ law region. For composites and adhesives, the strain energy release rate G is more commonly used than K, but much discussion still remains in the expression for G [28,29].

In the study of fatigue behavior of adhesives, not only were the fatigue properties of the adhesive itself investigated, but also the effects of layer thickness [30,31,32,33,34,35], load level [28,36] and surface treatment [37,38,39] on FCG. However, it should be noted that most studies on adhesive fatigue deal with epoxies. Nowadays, many other polymers have also been used as adhesives. In particular, structural acrylic adhesives are attracting much attention as room-temperature curable adhesives with strength and toughness comparable to those of epoxies. Because ambient-temperature curing adhesives do not require additional equipment for curing and there are no restrictions on the size or location of the products to be bonded, they have been used to bond large structures such as ships, turbine blades, and door panels, or to assemble/repair on-site. However, there have been relatively few papers dealing with FCG in structural acrylic adhesives [40,41,42,43].

To select an optimal bond-line thickness and indicate the sensitivity of fracture toughness to thickness variation, it is important to understand the effect of bond-line thickness on the fracture behavior under various loading conditions. In the case of epoxy adhesives, many studies reported that the fracture toughness and the fatigue resistance are highly dependent on thickness [30,31,32,33,34,35,44,45,46,47] but that the effect of the thickness on FCG is not consistent [35]. In addition, the importance of a damage zone size in fatigue properties was suggested [34]. Under the quasi-static loading, not only the adhesive thickness but also the surface micro-patterns change the fracture process zone area that leads to an increase in the fracture energy [48]. In the case of structural acrylic adhesives, it has been reported that the fracture toughness becomes larger with larger bond-line thickness under a quasi-static condition and it approached the intrinsic work of the fracture $G_{0}$ with the decrease in the plastic zone size under impact loading [49]. Further understanding of fracture behavior through experiments under cyclic loading is crucial for increasing the reliability of the joints bonded with structural acrylic adhesives.

In the present work, the effect of bond-line thickness of the structural acrylic adhesive on fatigue resistance was discussed by conducting fatigue double cantilever beam (DCB) tests for mode I fractures. Five different thickness samples (0.15 to 0.82 mm) were prepared and FCG behavior was investigated under the same loading condition. In addition, the effects of R-ratio and initial crack length on FCG were also investigated for two selected bond-line thicknesses (0.15 and 0.60 mm). Crack propagation per cycle was calculated using a compliance-based method and fatigue crack behavior was investigated. Then, differences in FCG in accordance with bond-line thickness and fatigue test conditions were discussed.

## 2. Experimental

### 2.1. Materials

Carbon steel (S50C) was used as the substrate. A two-part acrylic-based adhesive (Hardloc C355-20 A/B, Denka Co., Ltd., Tokyo, Japan), which is a second-generation acrylic (SGA) adhesive, was used for bonding the substrates. Microscale sea-island structures are formed in SGA due to phase separation at the initial stage of the curing process [50]. Owing to the difference in stiffness between the sea part and the island part, the SGA adhesives can efficiently generate micro-cracks during plastic deformation and provide excellent toughness, which leads to a large process zone around the crack [51].

### 2.2. Specimen Preparation

**Figure 1:**
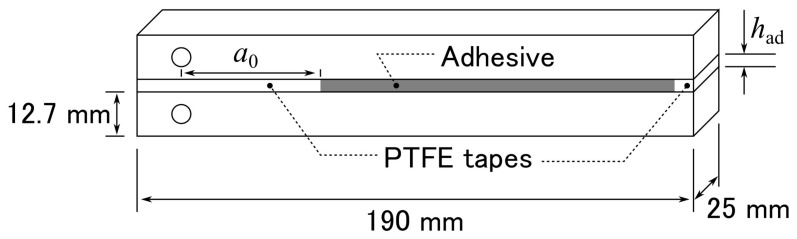
Schematic of DCB specimen geometry.

The specimen geometry is shown in Figure 1. The surfaces of the substrates were sandblasted with Al_2_O_3_ grit as an abrasive medium and wiped with acetone prior to bonding. The adhesive thickness was controlled by inserting a polytetrafluoroethylene (PTFE) tape at the front and back of the adhesive layer. After the substrates were bonded together, they were cured initially at approximately 24 °C for 24 h, and then post-cured at 60 °C for 2 h. The substrate thickness was measured after sandblasting and specimen thickness after bonding; then, the adhesive thickness was inferred using the substrate and specimen thicknesses. For the reliability of the experiments, the number of trials is important. However, fatigue tests generally take much longer than quasi-static tests, and the number must inevitably be limited. Therefore, in this study, instead of increasing the number of samples in each condition, we gradually changed the conditions and prepared one sample for each condition to evaluate overall trends.

### 2.3. Fatigue Testing

**Figure 2:**
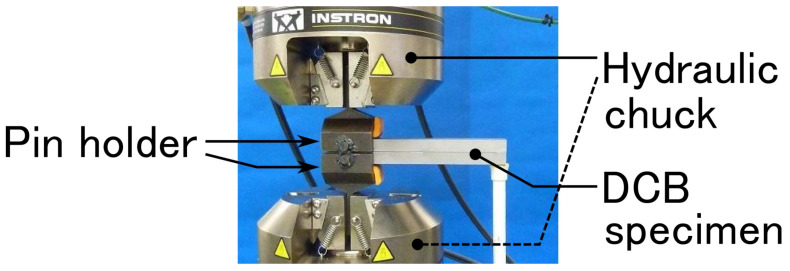
Photograph of the fatigue test setup.

A servo-hydraulic fatigue testing machine (8800 series with a load cell capacity of 100 kN, Instron Japan Co., Ltd., Kanagawa, Japan) was used. The specimen was attached to a hydraulic chuck via a pin holder, as shown in Figure 2. The DCB fatigue tests were performed at a cyclic frequency of 10 Hz under displacement control at room temperature (approximately 24 °C). Maximum and minimum displacements are denoted as $\delta_{\max}$ and $\delta_{\min}$. After $10^{6}$ cycles of the fatigue test, a quasi-static fracture test was conducted with a displacement speed of 1 mm/min using the same machine. The adhesive bond-line thickness, $h_{\mathrm{ad}}$, was varied holding other parameters in a fixed state, as shown in Table 1, and the effect of thickness on fatigue characteristics was investigated. According to a previous study [49], a linear increase in fracture toughness with respect to adhesive thickness was predicted for the SGA adhesive used when $h_{\mathrm{ad}}<1$ mm. Therefore, the adhesive thickness was gradually changed within a range of $0.15\leq h_{\mathrm{ad}}<1$ mm. In addition, the effects of the R-ratio, R, and initial crack length, $a_{0}$, on the fatigue behavior were also investigated for two specific bond-line thicknesses, as shown in Table 2.

### 2.4. Data Reduction Approach

Accurate measurement of the crack length is always difficult in the fatigue and quasi-static fracture tests of adhesives. In particular, for highly ductile adhesives, a large fracture process zone (FPZ) generated at the crack tip must be taken into account in fracture energy calculation. A compliance-based crack estimation method has been established based on linear elastic fracture mechanics (LEFM) for the adhesively bonded DCB specimens, therefore eliminating the need to measure the crack length and FPZ length [52,53,54]. Neglecting the shear effect of the beam deflection, the equivalent crack length, a, which expresses the crack length including the FPZ length, can be calculated as:

(1) $$a={\left(\frac{3}{2}EIC\right)}^{1/3}$$

where E is the substrate modulus, I is the moment of inertia of the substrate cross-section, and C is the compliance that was obtained from the gradient of the load-displacement (P—δ) curve for each cycle. The fracture energy is then obtained as follows:

(2) $$G=\frac{P^{2}a^{2}}{bEI}$$

where P is the applied load and b is the width of the specimen, respectively. For the fatigue tests, the fracture energy range, ΔG, was obtained from the maximum and minimum values, $G_{\max}$ and $G_{\min}$, for each cycle:

(3) $$\Delta G=G_{\max}-G_{\min}$$

**Figure 3:**
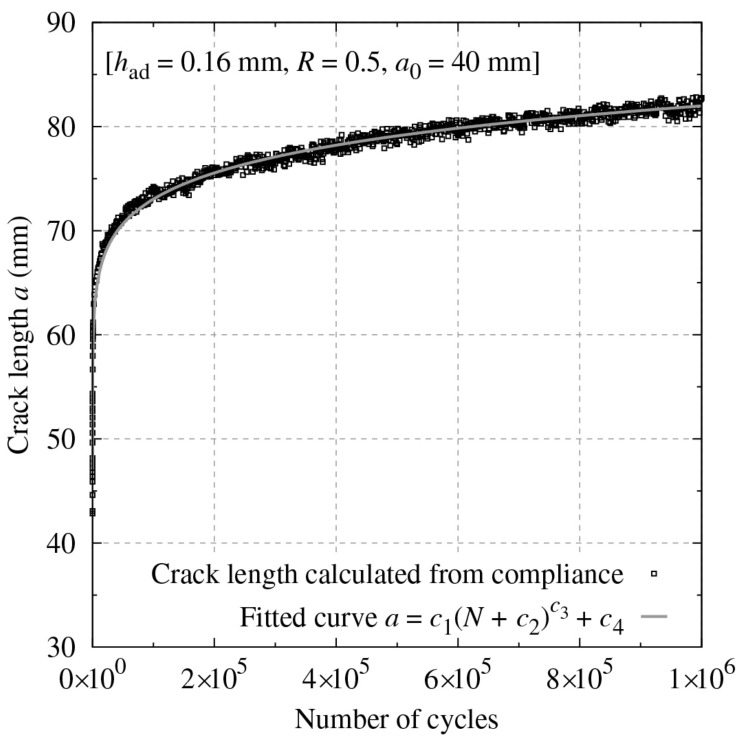
Example of a power-like law fit to the relation between a and N.

To calculate the crack growth rate per cycle da/dN while avoiding data scattering, a power-like law was adopted:

(4) $$a=c_{1}{\left(N+c_{2}\right)}^{c_{3}}+c_{4}$$

where $c_{1},\;c_{2},\;c_{3}$ and $c_{4}$ are constants obtained by the least-squares fit for each test and N is the number of cycles [28]. Figure 3 shows an example of the fitted results. Then, da/dN was calculated by differentiating Equation (4):

(5) $$\frac{da}{dN}=c_{1}c_{3}{\left(N+c_{2}\right)}^{c_{3}-1}$$

## 3. Results and Discussion

### 3.1. Adhesive Bond-Line Thickness Effect

**Figure 4:**
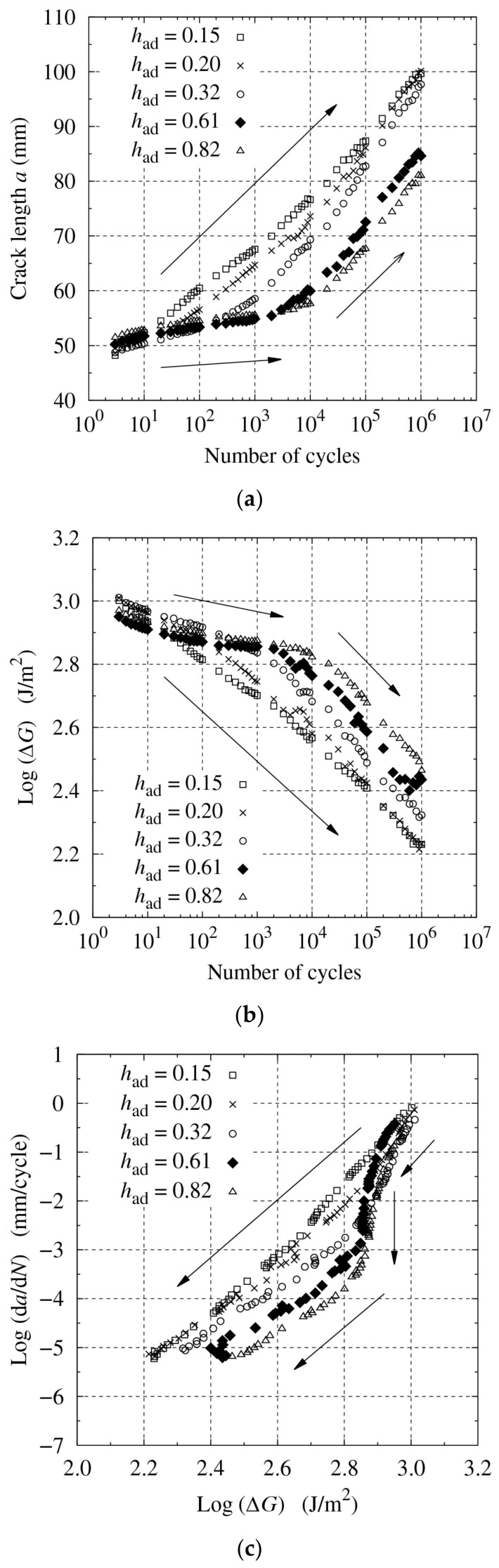
Fatigue test results for R=0.2 and $a_{0}=40$ mm and varying bond-line thickness: (**a**) a versus logN, (**b**) logΔG versus logN, and (**c**) logda/dN versus logΔG.

The results of the fatigue tests with varying bond-line thicknesses are shown in Figure 4. A cohesive fracture was observed for all thickness configurations. As the initial crack length and loading conditions were the same for all the tests, the initial reactions were similar regardless of the bond-line thickness. However, as the number of cycles increased, the advantage of a thicker bond line clearly revealed. The gradient changed around the middle for $h_{\mathrm{ad}}\geq 0.32$ mm, despite the linear increase in a and linear decrease in logΔG against logN for $h_{\mathrm{ad}}=0.15$ and 0.20 mm (see Figure 4a,b).

In the low-cycle range ($N<10^{4}$), the crack propagation speed was slower with a thicker bond line, which suggested that the fatigue crack resistance was greater with a thicker bond line under the same loading conditions. Conversely, in the high-cycle range ($N>10^{4}$), the gradients of a versus logN and that of logΔG versus logN were similar regardless of the bond-line thickness. Thus, the gradient of logda/dN versus logΔG became also similar for $da/dN<10^{-3}$ mm/cycle, as shown in Figure 4c. The linear range of logda/dN versus logΔG is named the Paris’ law region. Therefore, the full-cycle range for $h_{\mathrm{ad}}=0.15$ and 0.20 mm belonged to the Paris’ law region, but only the high-cycle range for $h_{\mathrm{ad}}\geq 0.32$ mm.

**Figure 5:**
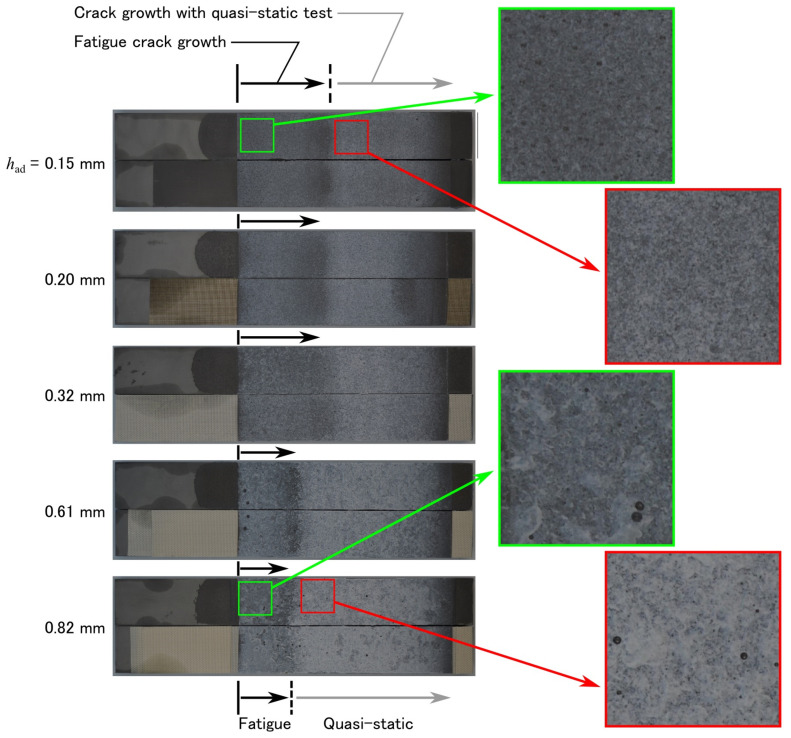
Fractured surfaces after the fatigue and quasi-static tests.

**Figure 6:**
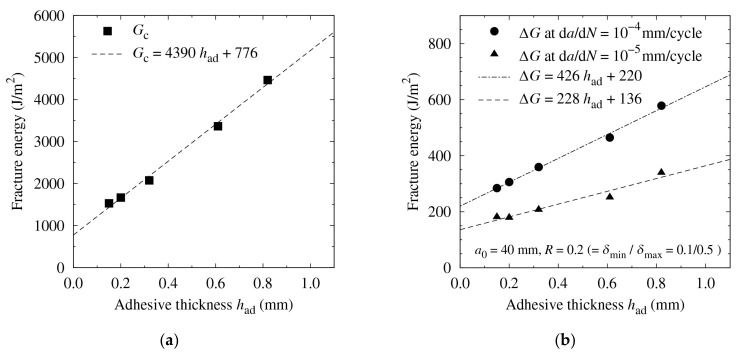
Effect of bond-line thickness on the fracture energy for (**a**) static fracture tests and (**b**) fatigue tests.

Owing to the different FCG behavior in the low-cycle range, ΔG was larger with a thicker bond line even after the high-cycle fatigue; therefore, the superiority of the thickness remained. Visual observation of the fracture surfaces revealed that the fatigue crack growth became shorter as the adhesive layer became thicker, as shown in Figure 5. Finally, the fracture energy was plotted against the bond-line thickness for the quasi-static fracture test in Figure 6a and the fatigue test in Figure 6b. In all cases, the fracture energy increased with an increase in thickness. In many cases of toughened epoxy adhesives, the fracture energy stopped increasing at a certain thickness, generally less than 1 mm [44,55]. However, with some types of structural acrylic and structural polyurethane adhesives, the fracture energy continued to increase even after the thickness exceeded 1 mm [49,56]. The experimental results revealed that this tendency was maintained even during the fatigue tests.

### 3.2. R-Ratio and Initial Crack Length Effects

The fatigue characteristics changed with the bond-line thickness when the R-ratio and initial crack length were held constant. However, it is also important to investigate the fatigue characteristics under different test conditions. Thus, two bond-line thicknesses that showed different fatigue characteristics under the same loading conditions were selected and the effects of the test conditions on the fatigue behavior were investigated.

**Figure 7:**
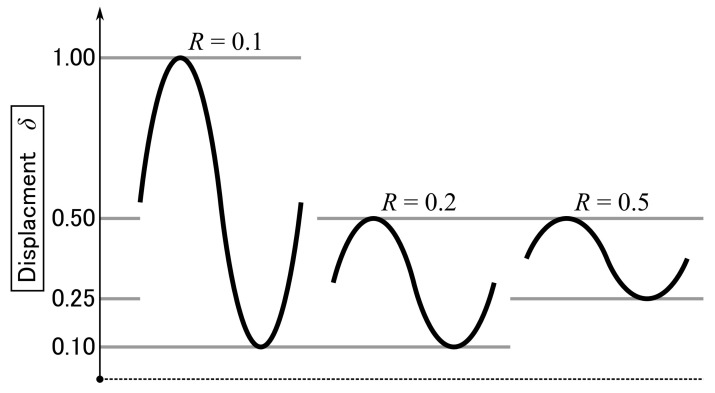
Schematic of the sinusoidal displacement input with the different R-ratios.

**Figure 8:**
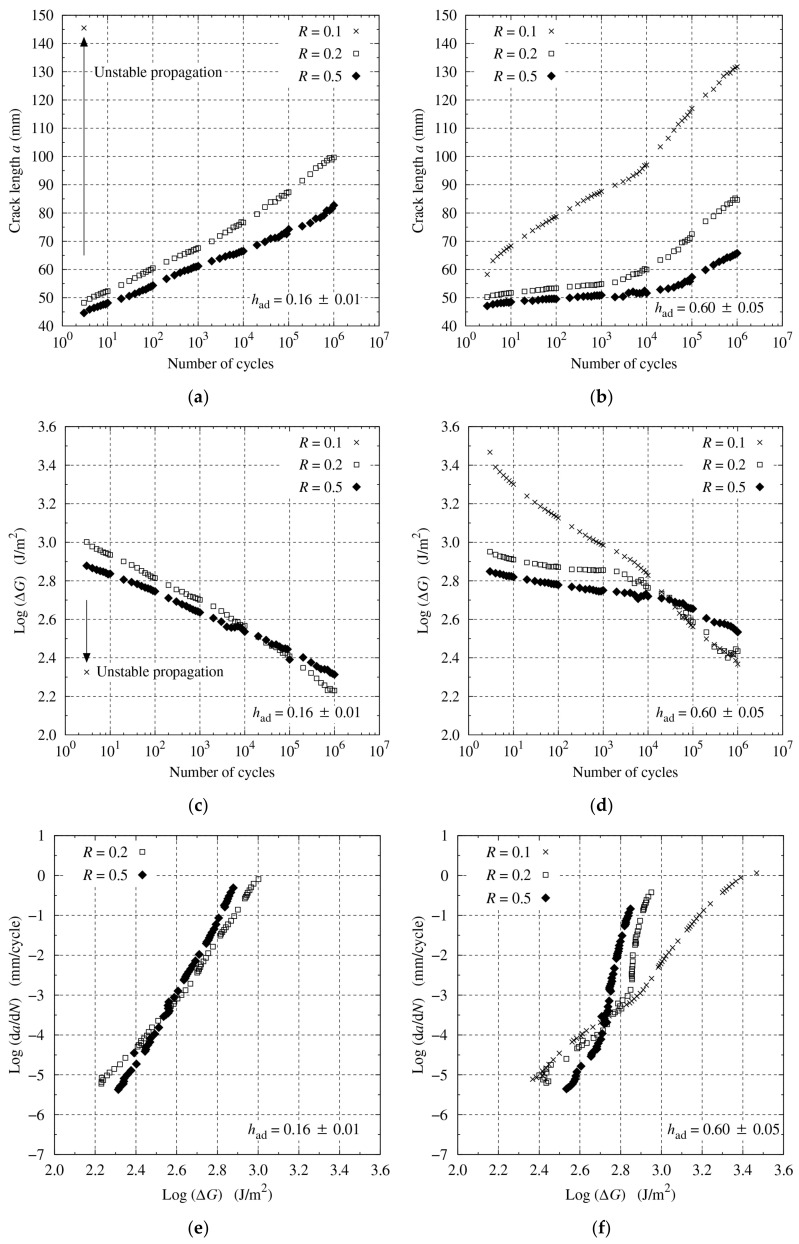
Fatigue test results for $a_{0}=40$ mm and varying R: (**a**,**b**) a versus logN; (**c**,**d**) logΔG versus logN; and (**e**,**f**) logda/dN versus logΔG, for $h_{\mathrm{ad}}=0.16\pm 0.01$ and 0.60±0.05 mm, respectively.

To vary the R-ratio, the maximum displacement $\delta_{\max}$ was increased from 0.5 to 1.0 mm for R=0.1 and the minimum displacement $\delta_{\min}$ from 0.10 to 0.25 mm for R=0.5, as shown in Figure 7. The results of the fatigue tests are presented in Figure 8. For the thin bond-line specimen, R=0.1 was too high to gradually propagate the crack and unstable crack propagation was observed at the initial stage of the test. Consequently, the test was stopped. For the remainder of the tests, the crack continuously propagated through the fatigue tests, although the crack propagated faster with a smaller R (see Figure 8a,b). The fracture energy range, ΔG, was smaller at the initial stage with a larger R because of larger $G_{\min}$ but decreased more slowly with a larger R (see Figure 8c,d). Therefore, the final ΔG was the largest at R=0.5. Conversely, a small difference in ΔG was observed in the high-cycle region for R=0.1 and R=0.2, with the thick bond-line specimen. The relationship between logda/dN and logΔG was linear across the whole range, but the gradient differed with the thin bond-line specimen (see Figure 8e). Conversely, with the thick bond-line specimen, it was almost linear across the whole range for R=0.1, but only in the high-cycle range for R=0.2 and 0.5 (see Figure 8f).

**Figure 9:**
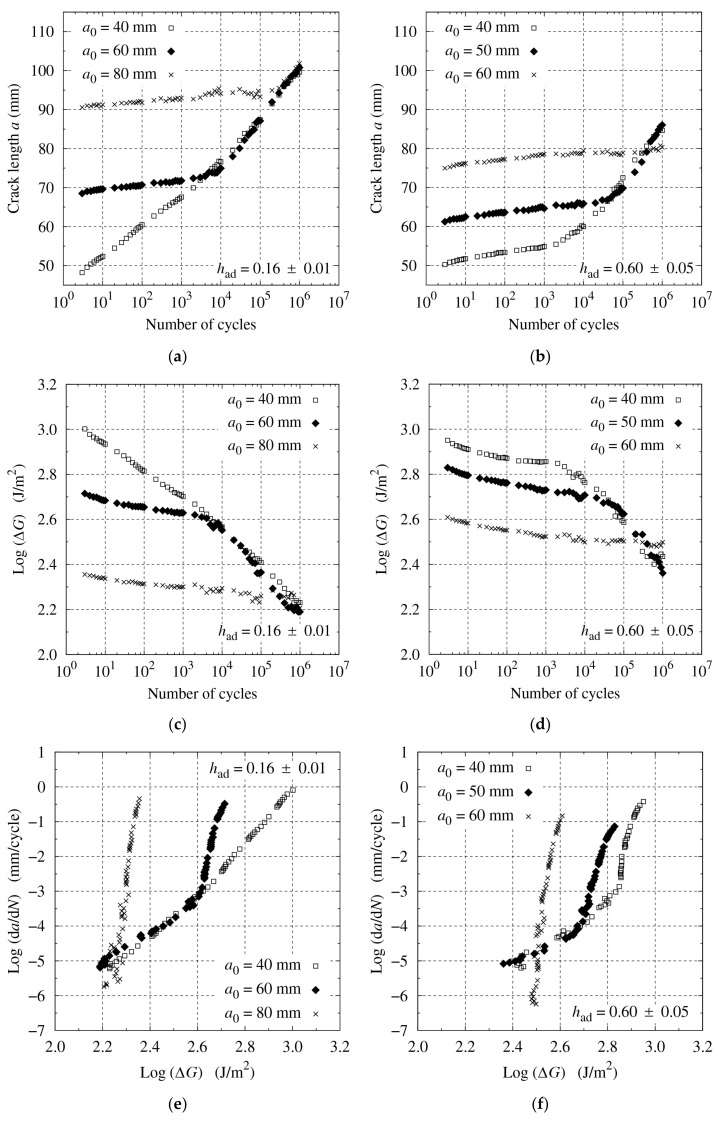
Fatigue test results for R=0.2 and varying $a_{0}$: (**a**), (**b**) a versus logN; (**c**), (**d**) logΔG versus logN; and (**e**), (**f**) logda/dN versus logΔG, for $h_{\mathrm{ad}}=0.16\pm 0.01$ and 0.60±0.05 mm, respectively.

Figure 9 shows the fatigue test results for varying initial crack lengths. The crack propagated slower and ΔG decreased less with a longer $a_{0}$ at the initial stage. After a sufficient number of cycles to reach the Paris’ law region, however, the crack propagation accelerated, and the fatigue characteristics became similar regardless of the initial crack length. In addition, when the initial crack was too long, a clear Paris’ law region did not appear even after $10^{6}$ cycles.

Comparing the ΔG of $h_{\mathrm{ad}}=0.16$ and $h_{\mathrm{ad}}=0.60$ after $10^{6}$ cycles (Figure 8c,d and Figure 9c,d), we found that the fatigue resistance was always higher when the bond line was thicker, regardless of the R-ratio and initial crack length. In particular, it was very interesting to be able to improve the joint that fractured with a few loading cycles to withstand $10^{6}$ vibrations under the same loading condition by simply changing the bond-line thickness.

### 3.3. Effect of Initial Load Level on Fatigue Crack Growth Behavior

Table 3 and Table 4 summarize the results. The fracture energy range ΔG was normalized by the critical fracture energy $G_{c}$. The initial maximum load measured in the fatigue tests was normalized by the initial maximum load in the quasi-static fracture tests that is theoretically calculated using the measured initial compliance and $G_{c}$. The normalized load was expressed as a maximum load level in percentage.

**Figure 10:**
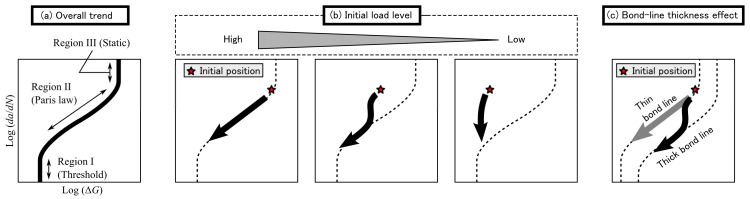
Schematics of fatigue fracture diagram as a relation between logda/dN and logΔG with (**a**) overall trend, (**b**) different initial load levels, and (**c**) different bond-line thicknesses.

Owing to the high-cycle fatigue, ΔG was reduced to approximately 1/10 of $G_{c}$ regardless of the initial load level. Conversely, a difference was observed, especially in the relations between logda/dN and logΔG. Therefore, the characteristics of the FCG behavior of the structural acrylic adhesive were categorized by the initial load level, as shown in Figure 10. A high load level ($\Delta G_{\mathrm{initial}}/G_{c}>0.5$ and/or maximum load level over 80%) was required for the Paris’ law region to appear in the low-cycle fatigue. Thus, the initial conditions at the high load level were considered to be on or near the FCG curve, as shown in Figure 10b. At a moderate initial load level, the fracture energy seemed to achieve the threshold value in the low-cycle region, but the fracture energy started decreasing again in the high-cycle region. The temporary threshold-like value in the low-cycle region was much smaller than $G_{c}$ and changed depending on the loading conditions. Therefore, we must be careful not to consider the temporarily stagnant value at low-cycle fatigue as the threshold. At a low initial load level ($\Delta G_{\mathrm{initial}}/G_{c}<0.2$ and/or maximum load level under 40%), almost no fatigue crack propagation was observed because the $\Delta G_{\mathrm{initial}}$ was too small. Therefore, applying the appropriate initial load is important for the appearance of Paris’ law region. In addition, the initial load level decreased with increasing the bond-line thickness when the loading condition was the same, but the initial position of logda/dN versus logΔG was kept close. Thus, the results from varying the bond-line thickness indicate a downward shift on the FCG curve (see Figure 10c).

## 4. Conclusions

The effect of adhesive bond-line thickness on fatigue toughness for mode I loading was investigated by conducting fatigue double cantilever beam (DCB) tests for a structural acrylic adhesive. The fatigue tests were conducted under displacement control with a frequency of 10 Hz. Giving the same fatigue load and varying the bond-line thickness, the initial ΔG was the same regardless of the thickness. However, in the low-cycle region (cycle number less than 10^4^), a difference in the fatigue crack growth (FCG) behavior was observed depending on the bond-line thickness. With an increase in the cycle number, a linear relationship between logda/dN and logΔG—i.e., Paris’ law region—was observed, regardless of the thickness and the gradient of the FCG curve became similar. As a result, when comparing the results at the same logda/dN, the fracture toughness was higher with thicker bond-line specimens, which was the same tendency as found in the quasi-static test results. Although it was revealed that the condition for the Paris’ law region to appear was highly dependent on the test conditions, especially the initial load level, the superiority of the high-bond-thickness samples was maintained across different test conditions even when changing the R-ratio and initial crack length. Therefore, when using the structural acrylic adhesive in a vibrating or cyclic loading environment, a thicker adhesive layer is preferred from the viewpoint of improving fatigue resistance. However, a clear threshold was not observed at $10^{6}$ cycles. Furthermore, in real usage, the joints are exposed to different environments, such as high temperature, high humidity, and UV irradiation. In recent years, life prediction by numerical analysis has been highly advanced, and shorter and more sophisticated analyses will be able to be performed by combining these with experiments. Therefore, it is expected that the actual durability will be confirmed in the future, considering its further exposure to external influences and harsher environments.

## Figures and Tables

**Table 1:** The test conditions for varying adhesive bond-line thickness.

$h_{\mathrm{ad}}\;(\mathrm{mm})$	$\delta_{\min}/\delta_{\max}\;\left(\mathrm{mm}\right)\;\left(=R\right)$	$a_{0}\;(\mathrm{mm})$
0.15	0.10/0.50 (=0.2)	40
0.20	0.10/0.50 (=0.2)	40
0.32	0.10/0.50 (=0.2)	40
0.61	0.10/0.50 (=0.2)	40
0.82	0.10/0.50 (=0.2)	40

**Table 2:** The test conditions for varying R-ratio and initial crack length.

$h_{\mathrm{ad}}\;(\mathrm{mm})$	$\delta_{\min}/\delta_{\max}\;\left(\mathrm{mm}\right)\;\left(=R\right)$	$a_{0}\;(\mathrm{mm})$
0.16 ± 0.01	0.10/1.00 (=0.1)	40
	0.10/0.50 (=0.2) ^1^	40
	0.25/0.50 (=0.5)	40
	0.10/0.50 (=0.2)	60
	0.10/0.50 (=0.2)	80
0.60 ± 0.05	0.10/1.00 (=0.1)	40
	0.10/0.50 (=0.2) ^1^	40
	0.25/0.50 (=0.5)	40
	0.10/0.50 (=0.2)	50
	0.10/0.50 (=0.2)	60

^1^ The same specimen as in Table 1.

**Table 3:** Fatigue test results compared to a quasi-static test varying the bond-line thickness.

$h_{\mathrm{ad}}$	R	$a_{0}$ (mm)	a (mm) after 10^6^ cycles	$\frac{\Delta G_{\mathrm{initial}}}{G_{c}}$	$\Delta G/G_{c}$ at $da/dN=10^{-5}$ (mm/cycle)	Maximum Load Level (%)
0.15	0.2	40	99.7	0.66	0.12	83
0.20	0.2	40	100	0.61	0.11	80
0.32	0.2	40	97.7	0.49	0.10	72
0.61	0.2	40	84.6	0.27	0.08	52
0.82	0.2	40	81.0	0.21	0.08	46

**Table 4:** Fatigue test results compared to a quasi-static test varying the R-ratio and initial crack length.

$h_{\mathrm{ad}}$	R	$a_{0}$ (mm)	a (mm) after 10^6^ cycles	$\frac{\Delta G_{\mathrm{initial}}}{G_{c}}$	$\Delta G/G_{c}$ at $da/dN=10^{-5}$ (mm/cycle)	Maximum Load Level (%)
0.15 ± 0.02	0.1	40	N/A	>1	N/A	>100
	0.2	40	99.7	0.67	0.12	83
	0.5	40	82.7	0.50	0.16	79
	0.2	60	101	0.35	0.11	59
	0.2	80	102	0.15	0.13	40
0.60 ± 0.05	0.1	40	132	0.88	0.08	93
	0.2	40	84.6	0.27	0.08	52
	0.5	40	65.8	0.21	0.11	49
	0.2	50	86.0	0.22	0.09	47
	0.2	60	80.3	0.12	0.10	36

## Data Availability

Data is contained within the article.

## References

[1] ASTM D3166-99(2020) (2020). Standard Test Method for Fatigue Properties of Adhesives in Shear by Tension Loading (Metal/Metal).

[2] ISO 9664:1993 (1993). Adhesives—Test Methods for Fatigue Properties of Structural Adhesives in Tensile Shear.

[3] ASTM D3433-99(2020) (2020). Standard Test Method for Fracture Strength in Cleavage of Adhesives in Bonded Metal Joints.

[4] ISO 25217: 2009 (2009). Adhesives—Determination of the Mode I Adhesive Fracture Energy of Structural Adhesive Joints Using Double Cantilever Beam and Tapered Double Cantilever Beam Specimens.

[5] Blackman B.R.K., Kinloch A.J., Paraschi M., Teo W.S. (2003). Measuring the mode I adhesive fracture energy, GIC, of structural adhesive joints: The results of an international round-robin. Int. J. Adhes. Adhes..

[6] Shimamoto K., Sekiguchi Y., Sato C. (2016). Mixed mode fracture toughness of adhesively bonded joints with residual stress. Int. J. Solids Struct..

[7] Sekiguchi Y., Yamagata Y., Sato C. (2017). Mode I fracture energy of adhesive joints bonded with adhesives with different characteristics under quasi-static and impact loading. J. Adhes. Soc. Jpn..

[8] Komatsu K., Sekiguchi Y., Ihara R., Tatsumi A., Sato C. (2019). Experimental investigation of an adhesive fracture energy measurement by preventing plastic deformation of substrates in a double cantilever beam test. J. Adhes..

[9] Da Silva L.F.M., Campilho R.D.S.G. (2012). Advances in Numerical Modeling of Adhesive Joints.

[10] Álvarez D., Blackman B.R.K., Guild F.J., Kinloch A.J. (2014). Mode I fracture in adhesively-bonded joints: A mesh-size independent modelling approach using cohesive elements. Eng. Fract. Mech..

[11] Fernandes R.L., Campilho R.D.S.G. (2017). Testing different cohesive law shapes to predict damage growth in bonded joints loaded in pure tension. J. Adhes..

[12] Nunes F.A.A., Campilho R.D.S.G. (2018). Mized-mode fracture analysis of adhesively-bonded joints using the ATDCB test specimen. Int. J. Adhes. Adhes..

[13] Ramalho L.D.C., Campilho R.D.S.G., Belinha J., da Silva L.F.M. (2020). Static strength prediction of adhesive joints: A review. Int. J. Adhes. Adhes..

[14] Jablonski D.A. (1980). Fatigue crack growth in structural adhesives. J. Adhes..

[15] Kinloch A.J., Osiyemi S.O. (1993). Predicting the fatigue life of adhesively-bonded joints. J. Adhes..

[16] Jethwa J.K., Kinloch A.J. (1997). The fatigue and durability behaviour of automotive adhesives. Part I: Fracture mechanics tests. J. Adhes..

[17] Brunner A.J., Murphy N., Pinter G. (2009). Development of a standardized procedure for the characterization of interlaminar delamination propagation in advanced composites under fatigue mode I loading conditions. Eng. Fract. Mech..

[18] Azari S., Papini M., Schroeder J.A., Spelt J.K. (2010). Fatigue threshold behavior of adhesive joints. Int. J. Adhes. Adhes..

[19] Stelzer S., Brunner A.J., Argüelles A., Murphy N., Cano G.M., Pinter G. (2014). Mode I delamination fatigue crack growth in unidirectional fiber reinforced composites: Results from ESIS TC4 round-robins. Eng. Fract. Mech..

[20] Usman M., Pascoe J.A., Alderliesten R.C., Benedictus R. (2018). The effect of temperature on fatigue crack growth in FM94 epoxy adhesive bonds investigated by means of energy dissipation. Eng. Fract. Mech..

[21] Adamos L., Tsokanas P., Loutas T. (2020). An experimental study of the interfacial fracture behavior of Titanium/CFRP adhesive joints under mode I and mode II fatigue. Int. J. Fatigue.

[22] Jones R., Pneg D., Michopoulos J.G., Kinloch A.J. (2020). Requirements and variability affecting the durabilityof bonded joints. Materials.

[23] Khoramishad H., Crocombe A.D., Katnam K.B., Ashcroft I.A. (2010). Predicting fatigue damage in adhesively bonded joints using a cohesive zone model. Int. J. Fatigue.

[24] Eklind A., Walander T., Carlberger T., Stigh U. (2014). High cycle fatigue crack growth in mode I of adhesive layers: Modelling, simulation and experiments. Int. J. Fract..

[25] Pirondi A., Moroni F. (2019). Improvement of a cohesive zone model for fatigue delamination rate simulation. Materials.

[26] Rocha A.V.M., Akhavan-Safar A., Carbas R., Marques E.A.S., Goyal R., El-zein M., da Silva L.F.M. (2020). Numerical analysis of mixed-mode fatigue crack growth of adhesive joints using CZM. Theor. Appl. Fract. Mech..

[27] Paris P., Erdogan F. (1963). A critical analysis of crack propagation laws. J. Basic Eng..

[28] Simon I., Banks-Sills L., Fourman V. (2017). Mode I delamination propagation and R-ratio effects in woven composite DCB specimens for a multi-directional layup. Int. J. Fatigue.

[29] Rocha A.V.M., Akhavan-Safar A., Carbas R., Marques E.A.S., Goyal R., El-Zein M., da Silva L.F.M. (2020). Paris law relations for an epoxy-based adhesive. Int. Mech. Eng..

[30] Mall S., Ramanurthy G. (1989). Effect of bond thickness on fracture and fatigue strength of adhesively bonded composite joints. Int. J. Adhes. Adhes..

[31] Joseph R., Bell J.P., McEvily A.J., Liang J.L. (1993). Fatigue crack growth in epoxy/aluminum and epoxy/steel joints. J. Adhes..

[32] Xu X.X., Crocombe A.D., Smith P.A. (1996). Fatigue crack growth rates in adhesive joints tested at different frequencies. J. Adhes..

[33] Abou-Hamda M.M., Megahed M.M., Hammouda M.M.I. (1998). Fatigue crack growth in double cantilever beam specimen with an adhesive layer. Eng. Fract. Mech..

[34] Azari S., Papini M., Spelt J.K. (2011). Effect of adhesive thickness on fatigue and fracture of toughened epoxy joints—Part I: Experiments. Eng. Fract. Mech..

[35] Pascoe J.A., Zavatta N., Troiani E., Alderliesten R.C. (2020). The effect of bond-line thickness on fatigue crack growth rate in adhesively bonded joints. Eng. Fract. Mech..

[36] Shahverdi M., Vassilopoulos A.P., Keller T. (2012). Experimental investigation of R-ratio effects on fatigue crack growth of adhesively-bonded pultruded GFRP DCB joints under CA loading. Compos. Part A.

[37] Abel M.L., Adams A.N.N., Kinloch A.J., Shaw S.J., Watts J.F. (2006). The effect of surface pretreatment on the cyclic-fatigue characteristics of bonded aluminium-alloy joints. Int. J. Adhes. Adhes..

[38] Azari S., Papini M., Spelt J.K. (2010). Effect of surface roughness on the performance of adhesive joints under static and cyclic loading. J. Adhes..

[39] Bello I., Alowayed Y., Albinmousa J., Lubineau G., Merah N. (2021). Fatigue crack growth in laser-treated adhesively bonded composite joints: An experimental examination. Int. J. Adhes. Adhes..

[40] Jones R., Hu W., Kinloch A.J. (2015). A convenient way to represent fatigue crack growth in structural adhesives. Fatigue Fract. Eng. Mater. Struct..

[41] Kim H.B., Naito K., Oguma H. (2017). Fatigue crack growth properties of a two-part acrylic-based adhesive in an adhesive bonded joint: Double cantilever-beam tests under mode I loading. Int. J. Fatigue.

[42] Imanaka M., Ishii K., Hara K., Ikeda T., Kouno Y. (2018). Fatigue crack propagation rate of CFRP/aluminum adhesively bonded DCB joints with acrylic and epoxy adhesives. Int. J. Adhes. Adhes..

[43] Racha A.V.M., Akhavan-Safar A., Carbas R., Marques E.A.S., Goyal R., El-zein M., da Silva L.F.M. (2020). Fatigue crack growth analysis of different adhesive systems: Effects of mode mixity and load level. Fatigue Fract. Eng. Mater. Struct..

[44] Kinloch A.J., Shaw S.J. (1981). The fracture resistance of a toughened epoxy adhesive. J. Adhes..

[45] Da Silva L.F.M., de Magalhães F.A.C.R.G., Chaves F.J.P., de Moura M.F.S.F. (2010). Mode II fracture toughness of a brittle and a ductile adhesive as a function of the adhesive thickness. J. Adhes..

[46] Marzi S., Biel A., Stigh U. (2011). On experimental methods to investigate the effect of layer thickness on the fracture behavior of adhesively bonded joints. Int. J. Adhes. Adhes..

[47] Han X., Jin Y., da Silva L.F.M., Costa M., Wu C. (2020). On the effect of adhesive thickness on mode I fracture energy—An experimental and modelling study using a trapezoidal cohesive zone model. J. Adhes..

[48] Sun F., Pruncu C.I., Penchev P., Jiang J., Dimov S., Blackman B.R.K. (2020). Influence of surface micropatterns on the mode I fracture toughness of adhesively bonded joints. Int. J. Adhes. Adhes..

[49] Sekiguchi Y., Sato C. (2021). Experimental investigation of the effect of adhesive thickness on the fracture behavior of structural acrylic adhesive joints under various loading rates. Int. J. Adhes. Adhes..

[50] Hayashi A., Sekiguchi Y., Sato C. (2021). AFM observation of sea-island structure formed by second generation acrylic adhesive. J. Adhes..

[51] Kamiyama K., Mikuni M., Matsumoto T., Matsuda S., Kishi H. (2020). Crack growth mechanism on SGA adhesive joints. Int. J. Adhes. Adhes..

[52] De Moura M.F.S.F., Morais J.J.L., Dourado N. (2008). A new data reduction scheme for mode I wood fracture characterization using the double cantilever beam test. Eng. Fract. Mech..

[53] Sekiguchi Y., Katano M., Sato C. (2017). Experimental study of the mode I adhesive fracture energy in DCB specimens bonded with a polyurethane adhesive. J. Adhes..

[54] Sekiguchi Y., Hayashi A., Sato C. (2020). Analytical determination of adhesive layer deformation for adhesively bonded double cantilever beam test considering elastic-plastic deformation. J. Adhes..

[55] Lee D.-B., Ikeda T., Miyazaki N., Choi N.-S. (2004). Effect of bond thickness on the fracture toughness of adhesive joints. Trans. ASME.

[56] Banea M.D., da Silva L.F.M., Campilho R.D.S.G. (2015). The effect of adhesive thickness on the mechanical behavior of a structural polyurethane adhesive. J. Adhes..

